# Photoplethysmography-Based Machine Learning Approaches for Atrial Fibrillation Prediction: A Report From the Huawei Heart Study

**DOI:** 10.1016/j.jacasi.2021.09.004

**Published:** 2021-12-21

**Authors:** Yutao Guo, Hao Wang, Hui Zhang, Tong Liu, Luping Li, Lingjie Liu, Maolin Chen, Yundai Chen, Gregory Y.H. Lip

**Affiliations:** a Department of Pulmonary Vessel and Thrombotic Disease, Sixth Medical Center, Chinese PLA General Hospital, Beijing, China; b Department of Cardiology, Second Medical Center, Chinese PLA General Hospital, Beijing, China; c Tianjin Key Laboratory of Ionic-Molecular Function of Cardiovascular Disease, Tianjin Institute of Cardiology, Second Hospital of Tianjin Medical University, Tianjin, China; d Huawei Technologies Co, Shenzhen, China; e Liverpool Centre for Cardiovascular Sciences, University of Liverpool, Liverpool, United Kingdom; f Aalborg Thrombosis Research Unit, Department of Clinical Medicine, Aalborg University, Aalborg, Denmark

**Keywords:** artificial intelligence, atrial fibrillation, machine learning, risk prediction, AF, atrial fibrillation, AUC, area under receiver operating characteristic curve, ECG, electrocardiography, NPV, negative predictive value, PPG, photoplethysmography, PPV, positive predictive value, ROC, receiver operating characteristic

## Abstract

**Background:**

Current wearable devices enable the detection of atrial fibrillation (AF), but a machine learning (ML)–based approach may facilitate accurate prediction of AF onset.

**Objectives:**

The present study aimed to develop, optimize, and validate an ML-based model for real-time prediction of AF onset in a population at high risk of incident AF.

**Methods:**

A primary ML-based prediction model of AF onset (M1) was developed on the basis of the Huawei Heart Study, a general-population AF screening study using photoplethysmography (PPG)–based smart devices. After optimization in 554 individuals with 469,267 PPG data sets, the optimized ML-based model (M2) was further prospectively validated in 50 individuals with paroxysmal AF at high risk of AF onset, and compared with 72-hour Holter electrocardiographic (ECG) monitoring, a criterion standard, from September 1, 2019, to November 5, 2019.

**Results:**

Among 50 patients with paroxysmal AF (mean age 67 ± 12 years, 40% women), there were 2,808 AF events from a total of 14,847,356 ECGs over 72 hours and 6,860 PPGs (45.83 ± 13.9 per subject per day). The best performance of M1 for AF onset prediction was achieved 4 hours before AF onset (area under the receiver operating characteristic curve: 0.94; 95% confidence interval: 0.93-0.94). M2 sensitivity, specificity, positive predictive value, negative predictive value, and accuracy (at 0 to 4 hours before AF onset) were 81.9%, 96.6%, 96.4%, 83.1%, and 88.9%, respectively, compared with 72-hour Holter ECG.

**Conclusions:**

The PPG- based ML model demonstrated good ability for AF prediction in advance. (Mobile Health [mHealth] technology for improved screening, patient involvement and optimizing integrated care in atrial fibrillation; ChiCTR-OOC-17014138)

Atrial fibrillation (AF) is the most common cardiac rhythm disorder; because of its association with increased risk of stroke, heart failure, dementia, and death (1), efforts have been directed toward improving the detection of and screening for AF (2).

Screening for AF can be systematic or opportunistic, with the latter being more cost-effective, especially considering that patients at high risk of incident AF (eg, those with previous myocardial infarction, heart failure, chronic chest disease, or stroke) would normally attend clinical follow-up with health professionals. Various common and validated risk factors have been used to propose clinical risk prediction models for incident AF, but most of these models only have modest predictive value (3). Recent studies have shown that the clinical scores predicting AF recurrence after ablation have limited predictive ability (4). Nonetheless, clinical risk prediction scores can be used to identify high-risk subjects (eg, after stroke) who should be targeted for more intense screening efforts (5).

A clinical approach to AF screening has recently been complemented by various “smart” options for improving AF detection, including smart devices, wearable patches, and wearable devices, such as smartwatches linked to smartphones (6). Current wearable devices enable the detection of AF, but a machine learning (ML)–based approach may facilitate even more accurate prediction of incident AF. In the Huawei Heart Study, we conducted a population-based screening study of 187,912 individuals, where 0.23% received a “suspected AF” notification and 87.0% of those were confirmed as having AF, with a positive predictive value (PPV) of 91.6% (7). Thus, continuous home monitoring with smart device–based photoplethysmography (PPG) technology could be a feasible approach for AF screening. Nevertheless, it has not yet been investigated whether the prediction of AF onset can be improved with the use of the PPG signals from smart devices.

The objectives of this prespecified ancillary analysis from the Huawei Heart Study were to develop, optimize, and validate an ML-based model for predicting the onset of AF from normal sinus rhythm in patients at high risk of incident AF, eg, those with paroxysmal AF.

## Methods

### Population and data source

The development of the primary ML-based model relied on the AF screening phase, conducted from February 1, 2019, to July 31, 2019, of the Mobile Health Technology for Improved Screening, Patient Involvement, and Optimizing Integrated Care in Atrial Fibrillation (mAFA II) study (Huawei Heart Study), using Huawei smart devices (7). The design of the mAFA II study has been published (8). In brief, adults (age ≥18 years) could freely use an AF screening app with compatible Huawei smart devices based on PPG technology (Huawei Technologies Co) across China. Subjects aged <18 years and those unable to use a smartphone or a smart device were excluded. The diagnostic ability of the PPG algorithm for AF episodes (AF detection model [M0], developed by Huawei) and smart devices were validated before being used for the generaL population screening study (9,10).

**Figure 1:**
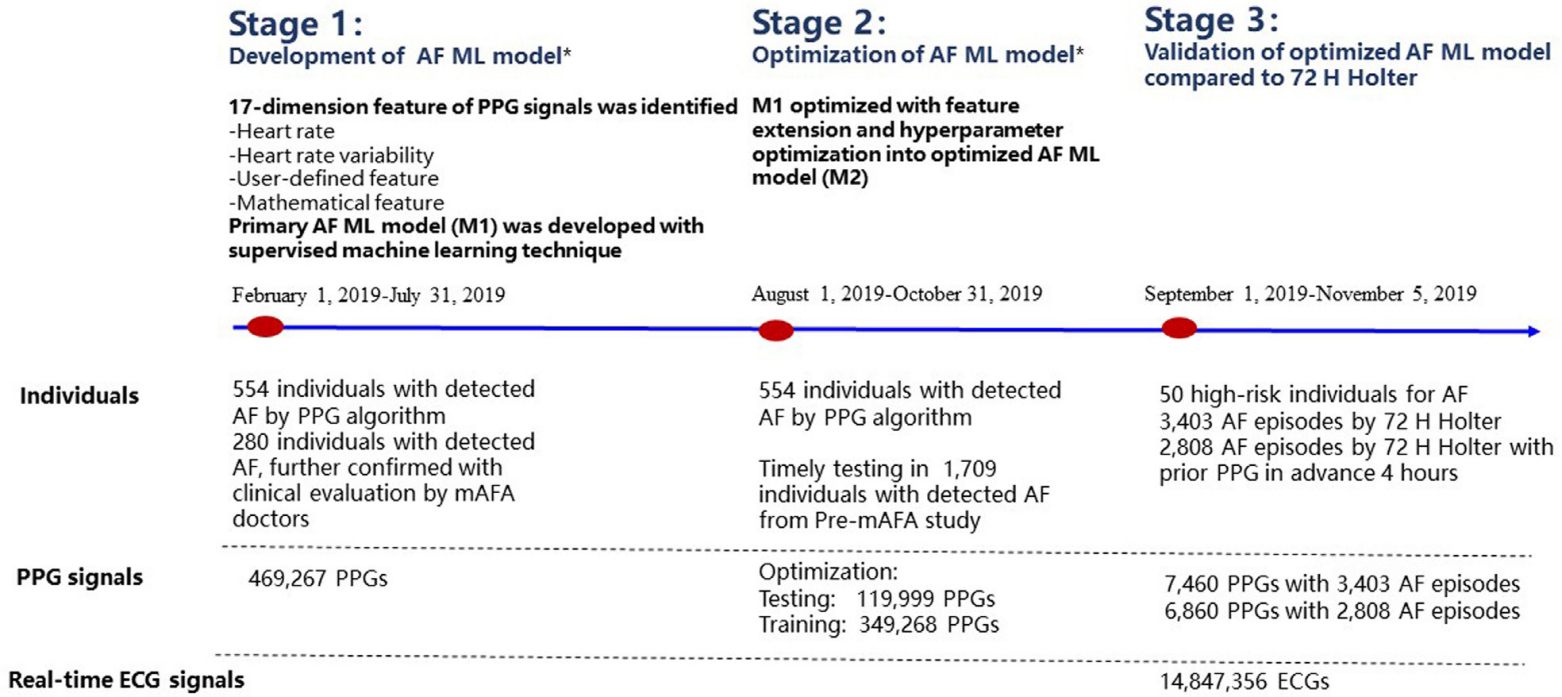
Flowchart of the Development, Optimization, and Validation of the Machine Learning–Based Atrial Fibrillation Prediction Model ∗Data were obtained from the Huawei Heart Study from February 1, 2019, to October 31, 2019, which was the first stage of the mAFA II (Mobile Health Technology for Improved Screening, Patient Involvement, and Optimizing Integrated Care in Atrial Fibrillation) study, using Huawei smart technology. AF = atrial fibrillation; ECG = electrocardiography; ML = machine learning; PPG = photoplethysmography.

The primary ML-based model for AF onset prediction (M1) was further optimized and tested into the optimized ML-based model for AF onset prediction (M2), which was compared with M0 and clinical diagnosis of AF episodes, from August 1, 2019, to October 31, 2019. M2, based on continuing PPG monitoring signals, was then prospectively validated in a population at high risk of AF onset and compared with continuous 72-hour Holter electrocardiography (ECG) monitoring from September 1, 2019, to November 5, 2019. The flowchart of the study protocol is shown in Figure 1. The study was approved by the Central Medical Ethics Committee of Chinese PLA General Hospital (approval number: S2017-105-02) and registered at www.chictr.org.cn (ChiCTR-OOC-17014138).

### Development of the primary ML-based AF prediction model (M1)

The continuing good quality of PPG signals and feature extraction were the key aspects for developing the ML model. Forty-eight–second raw waves in static state from the periodical measurements per 10 minutes were filtered, quality assessed, and then used for developing the model. After exploring XGBoost, Random Forest, Support Vector Machine, and Gradient Boosting Decision Tree with no significant difference, we decided to use XGBoost to develop the ML-based model in this study (Supplemental Figure 1).

Using radial PPG recording data of 273,743 subjects from the Pre-mAFA study with 554 participants to detect AF, we identified the following 17 features from the PPG data regarding: 1) heart rate: minimum value of all RR intervals (MinHR), mean value of all RR intervals (MeanHR), median value of all RR intervals (MedianHR), and skewness of all RR intervals (SkRR); 2) heart rate variability: coefficient of variation of all RR intervals (CVRR), standard deviation of all NN intervals (SDNN), SD ratio, and NN50 count divided by the total number of all NN intervals (pNN50); 2) user-defined features: probability of AF detection output (AFprob); and 4) mathematical features: sample entropy, Poincaré stepping, frequency domain, Shannon entropy, approximate entropy, difference, and PPG monitoring time. The features were chosen depending on the sensitivity of feature change and the extent of abnormal changes when closer to AF episodes.

### Optimization of the ML-based AF prediction model (M2)

The M1 was further optimized using feature extension (HRR features: combination of current RR features with history of RR features; CHRR features: HRR features with context information) (Supplemental Figure 1) and hyperparameter optimization on the basis of 554 individuals in whom AF was detected by the PPG algorithm. Of these, 266 detected AFs were further confirmed by clinical evaluation (including medical history and ECG) by a medical practitioner. The dataset of the 554 individuals with detected AF was randomly divided (3:1) into a training cohort and a testing cohort, with a total of 469,267 PPG signals available for the optimization of the model (Supplemental Figure 2). Test 1 included testing with 30,640 PPG signals for AF and 89,359 PPG signals for non-AF of 138 detected AF by PPG algorithm and further confirmed by doctors. Test 2 included testing with 30,640 PPG signals for AF and 89,359 PPG signals for non-AF from a total of 554 detected AF.

The real-time predictive ability of M2 for AF onset was tested among 1,709 individuals with detected AF from the pre-mAFA study from September 12, 2019, to December 10, 2019 (test 3) (Figure 1).

## Performance of the optimized model compared with 72-hour Holter ECG monitoring

Fifty individuals were enrolled to validate the predictive ability of M2 compared with 72-hour Holter ECG (test 4). Inclusion criteria were as follows adult age (≥18 years) and paroxysmal AF without current onset of AF. Exclusion criteria were age <18 years, presence of a pacemaker, and persistent/permanent AF. All subjects were required to monitor their pulse rhythm with a PPG-based wrist band while using 12-lead ECG monitoring continuously for 3 days. Thus, AF events were recorded simultaneously by the PPG algorithm (M0) and ECG.

**Figure 2:**
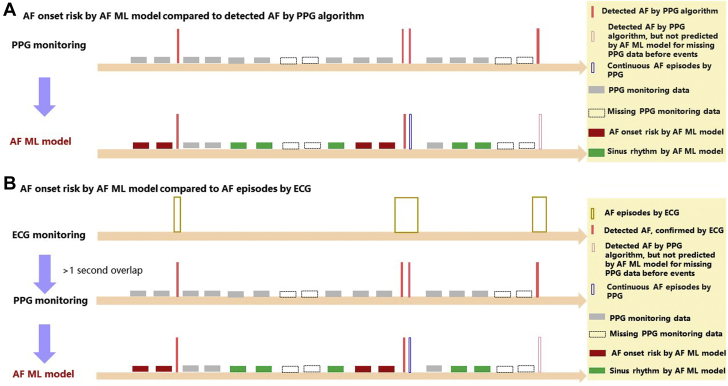
AF Onset Predicted With the ML-Based Model Compared With AF Detected by PPG algorithm and ECG **(A)** The risk of AF onset according to the AF ML model compared with AF detected by the PPG algorithm. **(B)** The risk of AF onset according to the AF ML model compared with AF episodes detected by ECG. Abbreviations as in Figure 1.

M2 was evaluated by comparing it with the PPG monitoring data and the 72-hour Holter ECG monitoring data (Figure 2). The “AF onset” events (AF onset lasting >30 seconds) predicted by M2, compared with AF detected by the PPG algorithm (M0) and AF episodes detected by ECG, are shown in Figure 2. AF detected by the PPG monitoring and AF episodes detected by the ECG monitoring needed to overlap at least 1 second to be considered the same AF event, taking detected AF and AF episodes as the “real” AF onset events (Figure 2).

### Statistical analysis

Continuous variables were tested for normality by means of the Kolmogorov-Smirnov test. Normally distributed data are presented as mean ± SD. Categoric variables are reported as n (%). The chi-square test was used to compare categoric variables. Receiver operating characteristic curve (ROC) analysis was used to evaluate the ML-based AF prediction model. The sensitivity, specificity, PPV, negative predictive value (NPV), and accuracy were also evaluated to analyze the performance of the model.

M1 was optimized to M2 by feature extension and hyperparameter optimization. M1 and M2 were both verified with the use of 2 randomly split data sets (Supplemental Figure 2). The area under the ROC curve (AUC), accuracy, F1 score, precision, and precision-recall graphs were used to analyze the predictive ability of M2 and compare it with that of M1. Using the DeLong equality test, we compared the AUC and C-statistic of M2 with those of M1. The false-positive rate was used to assess the predictive ability of the ML-based AF prediction model after optimization.

The real-time predictive ability of M2 for AF onset was further evaluated among 1,709 individuals with AF detected by the PPG algorithm (M0) on the basis of sensitivity and specificity. Sensitivity, specificity, PPV, NPV, and accuracy were used to evaluate M2 in the prospective cohort at high risk of AF onset with the use of AF episodes by 72-hour Holter and prior PPG monitoring data at the same time. The false-positive and false-negative rates of M2 in the validation cohort were further analyzed. The false positives were defined as PPG signals of AF occurrence predicted by M1 or M2 after 0 and 4 hours but not validated by a PPG (identified by M0) or an ECG with real AF occurrence. The false negatives were defined as PPG signals of no AF occurrence predicted by M1 or M2 after 0 and 4 hours but validated by a PPG or ECG with real AF occurrence.

### Sample size calculation of the prospective high-risk validation cohort

To ensure enough PPG signals to be used for M2 to predict AF in ECG test 4, the number of high-risk patients was estimated based on “real” AF detected by M0, considering the effective PPG monitoring signals before detected AF and the probability of AF occurrence.

Effective PPG monitoring signals were PPG monitoring signals over half of the monitoring time. Among those with effective PPG monitoring signals, there were 5.28 detected AF episodes per person per day, with a total of 29,976 AF detections among 126 suspected AF during 3 months from the Huawei Heart Study. Thus, a total of 15.84 detected AF episodes per person would be expected for 3 days, compared with the 72-hour Holter ECG.

The probability of AF occurrence was stratified from 10% to 100%, and at least 100 PPG signals were required to predict AF episodes in every risk stratum with type I error under 5% and power over 90%. When the probability of AF occurrence was over 80%, a total of 800 PPG signals were needed. We needed 50 high-risk individuals (800/15.84) to test the predictive ability of the M2 for AF onset at 4 hours before AF onset (Supplemental Figure 3).

The predictive ability of M2 for AF onset was validated in the high-risk cohort with the use of AF episodes by 72-hour Holter ECG with prior PPG 4 hours in advance. Sensitivity analysis of the predictive ability of M2 was performed with all AF episodes by 72-hour Holter ECG, with or without prior PPG 4 hours in advance.

A 2-sided *P* value of <0.05 was considered to be statistically significant. The 95% confidence intervals (CIs) were calculated using the Wilson score method without continuity correction. Statistical analysis was performed with the use of IBM SPSS Statistics version 25.0 and MedCalc 12.6.1.0.

## Results

### Development and optimization of the ML-based AF prediction model

**Figure 3:**
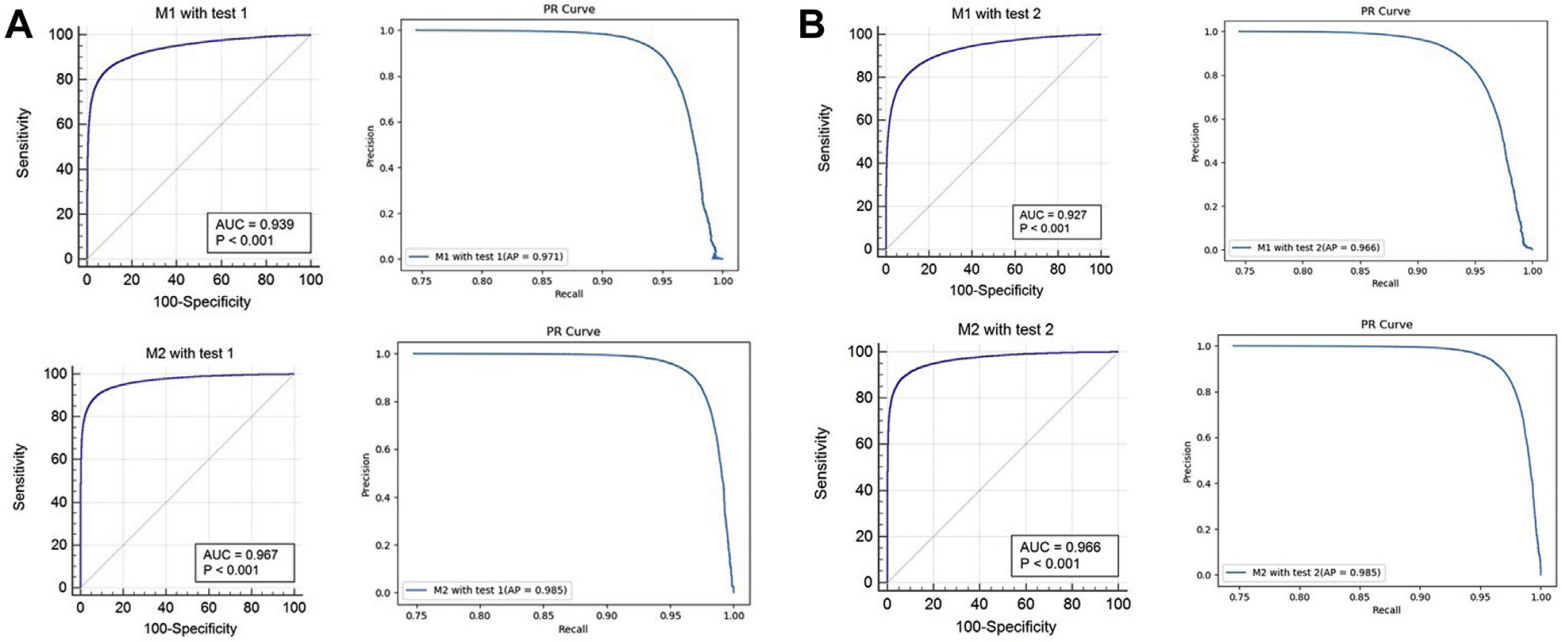
Receiver Operating Characteristic Curves and Precision-Recall Curves for AF Onset, 4 Hours in Advance **(A)** Primary AF ML model (M1) and optimized AF ML model (M2) with test 1. **(B)** Primary AF ML model (M1) and optimized AF ML model (M2) with test 2. AP = average precision; AUC = area under the receiver operating characteristic curve; other abbreviations as in Figure 1.

The best performance of M1 was achieved when predicting AF onset 4 hours before AF onset with a cutoff point of 0.5 (AUC: 0.94; 95% CI: 0.93-0.94), considering the underlying application of AI model in the clinical practice. M2 demonstrated an improved predictive ability in terms of the accuracy, precision, F1 score, and AUC (Table 1). The ROC and the precision-recall curve of M1 and M2 are presented in Figure 3. M2 was superior to M1 in predicting AF onset, with a difference between AUCs of 0.01-0.04, in 2 randomly split data sets (DeLong test, all *P* < 0.05) (Table 1, Supplemental Figure 4). Compared with M1, the false-positive rate of predicted AF onset with M2 was significantly reduced, by 1.19 ± 0.28 and 1.60 ± 0.44 in 2 randomly split data sets (all *P* < 0.05) (Supplemental Table 1).

**Table 1:** Predictive Ability of the Primary AF ML Model (M1) and the Optimized AF ML Model (M2)

	Test 1		Test 2	
	M1	M2	M1	M2
Accuracy	0.912 (0.910-0.913)	0.935 (0.933-0.936)	0.891 (0.890-0.893)	0.934 (0.933-0.936)
Precision	0.864 (0.860-0.868)	0.914 (0.910-0.917)	0.840 (0.836-0.844)	0.912 (0.909-0.916)
Sensitivity	0.775 (0.772-0.781)	0.821 (0.817-0.825)	0.730 (0.725-0.735)	0.813 (0.809-0.827)
Specificity	0.958 (0.957-0.959)	0.974 (0.973-0.975)	0.950 (0.948-0.951)	0.973 (0.972-0.974)
F1 score	0.818 (0.815-0.821)	0.865 (0.862-0.868)	0.781 (0.778-0.784)	0.865 (0.863-0.868)
AUC^a^	0.942 (0.933-0.943)	0.971 (0.970-0.981)	0.932 (0.923-0.935)	0.972 (0.971-0.982)

Values are % (95% confidence interval). Test 1: testing with 30,640 PPG signals for AF and 89,359 PPG signals for “non-AF” of 138 AF detections by the PPG algorithm, which were further confirmed by doctors. Test 2: testing with 30,640 PPG signals for AF and 89,359 PPG signals for “non-AF” from a total of 554 AF detections.

AF = atrial fibrillation; AUC = area under the receiver operating characteristic curve; ML = machine learning; PPG = photoplethysmography.

a Using the DeLong equality test, the diagnostic accuracy of M2 was compared with that of M1 (*P* < 0.001).

The optimized model (M2) was real-time tested among 1,709 individuals with detected AF during 3 months. During the 3-month testing period, the sensitivity and specificity of M2 for AF prediction 0 to 4 hours before AF onset were 0.78 and 0.93, respectively (Supplemental Figure 5).

The approaches of the ML-based AF prediction models 1 and 2 are shown in Supplemental Figure 6.

### Performance of the ML-based model compared with 72-hour Holter ECG monitoring

In 50 patients (mean age 67 ± 12 years, 40% women) with paroxysmal AF, the mean CHA_2_DS_2_-VASc risk score was 1.69 ± 1.34, and 7 of the 50 patients (14.0%) reported palpitation at enrollment (Supplemental Table 2). There were 3,403 AF events from 14,847,356 ECG monitoring data over 72 hours and 7,460 PPG monitoring data points over the same time period. Among those, there were 2,808 AF events with prior PPGs within 4 hours before onset, with a mean of 45.83 ± 13.9 PPG signals per subject per day.

Based on 2,808 AF events with prior PPGs, M2’s sensitivity, specificity, PPV, NPV, and accuracy for AF prediction at 0 to 4 hours before AF onset (with a cutoff at 0.5) were 81.9%, 96.6%, 96.4%, 83.1%, and 88.9%, respectively (Table 2). The AUC of M2 for predicting AF onset 4 hours in advance was 82.6% (95% CI: 81.6%-83.5%) (Supplemental Figure 6).

**Table 2:** Accuracy of the Optimized AF ML Model (M2) for AF Onset, With 2,808 AF Events by 72-Hour Holter and Prior PPG Monitoring

Time Before Event	Sensitivity	Specificity	PPV	NPV	Accuracy
1 h	0.916 (0.897-0.931)	0.955 (0.942-0.965)	0.945 (0.929-0.958)	0.931 (0.915-0.943)	0.937 (0.926-0.946)
2 h	0.886 (0.866-0.904)	0.963 (0.950-0.972)	0.956 (0.941-0.967)	0.902 (0.884-0.917)	0.926 (0.914-0.936)
3 h	0.848 (0.825-0.867)	0.965 (0.952-0.974)	0.960 (0.946-0.971)	0.863 (0.842-0.881)	0.906 (0.893-0.917)
4 h	0.819 (0.796-0.840)	0.966 (0.953-0.975)	0.963 (0.950-0.973)	0.831 (0.808-0.850)	0.889 (0.876-0.902)

Values are % (95% confidence interval).

NPV = negative predictive value; PPV = positive predictive value; other abbreviations as in Table 1.

The false-positive rate of AF prediction at 0 to 4 hours before AF onset with the use of M2 (with a cutoff at 0.5) was 5.58 (95% CI: 5.00-6.22). Of these false positives, more than 85% of the “real” events were atrial bigeminy, trigeminy, and atrial flutter (Supplemental Table 3).

Sensitivity analyses of the predictive ability of M2 with the use of 3,403 AF events with or without prior PPG within 4 hours are presented in Supplemental Table 4. The sensitivity and NPV of M2 with 3,403 AF events were slightly decreased, and the specificity and PPV were consistent with those of 2,809 AF events with prior PPG (Supplemental Table 4).

## Discussion

In a population-based screening cohort using PPG-based smart devices, our ML-based AF prediction model demonstrated high sensitivity, specificity, and PPV for AF prediction at 0 to 4 hours before AF onset. Such an ML approach may facilitate accurate prediction of AF onset and may help with choosing disease management options.

Clinical risk factors have been associated with incident AF (11), leading to the derivation and validation of clinical risk prediction scores for incident AF. Like most scores based on clinical factors, these scores have had only moderate predictive values (C-index: 0.6-0.7). Thus, improved risk prediction is needed to facilitate identification of high-risk subjects (eg, after stroke) who would be eligible for more intense AF screening efforts (12).

The “traditional” diagnosis of AF requires the use of a 12-lead ECG or 24-hour Holter ECG; however, the diagnostic ability of paroxysmal AF is associated with the frequency and duration of AF episodes. The longer and more frequent that AF occurs, the easier it is to be detected (13). Thus, an approach to monitor high-risk patients by combining clinical risk tools and ECG would be a reasonable cost-effective option to identify those who most need interventions. Smart technology may aid in this context. The use of comfortable smart wearables allows long-lasting continuous monitoring. In the Huawei Heart Study, nearly one-third of AF episodes were recorded after 2 weeks, without any complaints of skin irritation, anxiety, or device pressure (7). Similarly, in the Apple Watch Study, 75% of the longest detected AF episodes lasted no more than 6 hours (14). With the increasing availability of advanced monitoring technology and smart devices, the early detection/diagnosis of AF in the general population could be improved.

There has been much interest in the use of ML-based models for improving the diagnostic ability of ECG. For example, a deep neural network model has been developed on the basis of a single-lead, patch-based ambulatory ECG monitoring dataset with an average of 1.7 ECGs per patient, and it was validated on a test data set consisting of 328 ECG records collected from 328 unique patients; given that this model achieved an AUC >0.91 for all rhythm classes, including AF, it could improve the accuracy of algorithmic ECG interpretation (15). Similarly, facial recognition techniques based on deep learning can facilitate contact-free detection of AF from video data. Indeed, the ML-based approach may allow identification of AF in multiple individuals at the same time.

Moving beyond these initial studies, the real prediction of AF events (ie, before their onset), rather than identification/diagnosis of occurring AF events, has been explored with the help of artificial intelligence (AI) technology. Attia et al (16) has recently reported that AI-enabled 12-lead ECG acquired during normal sinus rhythm permits the prediction of AF in advance. With a mean of 3.6 ECGs per patient in the training, internal validation, and testing data sets, the AI-enabled ECG–identified AF model had an AUC of 0.87, sensitivity of 79.0%, specificity of 79.0%, F1 score of 32.0%, and overall accuracy of 79.0%. An ML-based model to predict incident AF during 6 months, which used 200 common electronic health record features, achieved an AUC of 0.80 and F1 score of 11.0% (17). Another ML-based AF prediction model was developed with the inclusion of clinical risk factors, such as demographic information, medical history, body mass index, and diastolic and systolic blood pressure. Compared with the clinical risk model using traditional methods (including logistic regression), the AUC of the ML-based model with clinical risk factors was higher (0.83 vs 0.72), achieving a PPV of 11.5% (vs 6.5%) (18).

However, rather than single-point measurements from prior studies as described above, an AI model based on dynamic monitoring data could provide much more accurate predictions of AF episodes. In the present prospective validation cohort, with a mean of 45.8 PPG signals per subject per day, our ML-based model had a sensitivity of 81.9%, specificity of 96.6%, F1 score of 82.0%, and overall accuracy of 88.9% for predicting AF onset. In the present analysis, we showed how ML-based models could be used to improve the prediction of AF from PPG-based smart devices. In both M1 and M2, the C-index was >0.9, with PPV of 96.4% and NPV of 83.1%. The ML-based AF prediction model with smart technology was able to make much more accurate predictions than traditional clinical risk assessment tools.

However, the extent to which AI technology improves predictive ability compared with traditional models possibly depends on the factors that are used to train the AI model. An AI model with “static” clinical risk factors might slightly or moderately improve the predictive ability with an AUC of 0.80-0.83 (17,18), but an AI model with more frequent, dynamic monitoring data could achieve an AUC of >0.90. Our study also demonstrated the application of ML to guide patient-level individualized early intervention. A data-driven “early warning” smart tool might not only identify AF episodes, but it could also predict AF onset in advance, thereby allowing physicians/patients more time to intervene and mitigate adverse outcomes. Of note, the predictive ability of the AI ML model is dependent on the PPG signals. Because a PPG-based wristband/watch provides continuous monitoring throughout the day, it would be less affected by AF burden compared with intermittent ECG monitoring.

This study raises many questions that need to be clarified. The feasibility of the use of AI models in clinical practice should be investigated, balancing the complexity of collecting more variables with the use of simple clinical factors in the logistic model. Another question arises as to which cases would a “highly predictive” AI model for AF onset be most suitable, and for which cases would a traditional clinical factor-based tool be better. In addition, it is important to understand what changes an AI model would bring to AF management. Large prospective cohort studies and randomized trials are needed to clarify these issues.

### Study limitations

First, we did not show possible impacts of the AI-supported AF prediction on clinical care. We only developed, optimized, and validated an ML-based model for predicting AF onset. In current clinical practice, AF onset is usually predicted by means of clinical risk scores with the use of traditional statistical models, with AUCs from 0.60 to 0.78 (12,19, 20, 21, 22). AI technology has been used to improve the diagnostic and predictive ability of traditional models, and it reached AUCs of 0.80 to 0.90 (16, 17, 18,23). In this study, the optimized ML-based model predicted AF onset 0 to 4 hours in advance with high sensitivity, specificity, and PPV, which demonstrates the feasibility of AI technology based on continuing PPG signals for real prediction, not just identification, of AF episodes. Further studies should explore the possibility of using AI ML models to predict AF onset in a longer time window before the actual AF onset so as to allow for the early intervention with upstream drugs or lifestyle behavior change to reduce the AF risk. Second, the ML algorithm needs to be accurate, and although the false-positive rate of 5% may be acceptable, 85% of the “real” events of false positives were atrial bigeminy, trigeminy, and atrial flutter, indicating the need to improve the discrimination of atrial arrhythmias other than AF. Nevertheless, the presence of frequent premature atrial contractions (PACs) or short runs of PACs may be an independent predictor of the development of atrial tachycardia and AF. How and when PACs transform into AF are questions that require further research to provide the basis for upstream interventions to prevent AF onset (24,25). The interpretability of methods applied to XGBoost models to improve the predictive ability (16,26,27), the comparison among different types of ML models, and introduction of clinical features into the ML model, will be further investigated. We developed, optimized, and validated an AF ML model in the present study, but its use in clinical practice will require evaluation in ongoing prospective studies.

## Conclusions

In a population-based screening cohort using PPG-based smart devices, an ML-based model demonstrated high sensitivity, specificity, and PPV for AF prediction at 0 to 4 hours before the actual AF onset. The ML-based approach may facilitate accurate prediction of AF onset (Central Illustration).

Perspectives **COMPETENCY IN MEDICAL KNOWLEDGE:** A machine-learning approach using smart device-based PPG technology facilitated accurate advance real-time prediction of AF onset. The artificial intelligence–based model with more frequent, dynamic monitoring data could achieve an AUC >0.90.

**TRANSLATIONAL OUTLOOK:** A data-driven “early warning” smart tool not only could identify/diagnose the AF events that did actually happen, but also could predict AF onset in advance, with much better predictive ability than traditional clinical factor–based tools. This would likely change AF prevention and management, but large prospective cohort studies and randomized trials are needed to address the future application.

**Central Illustration:**
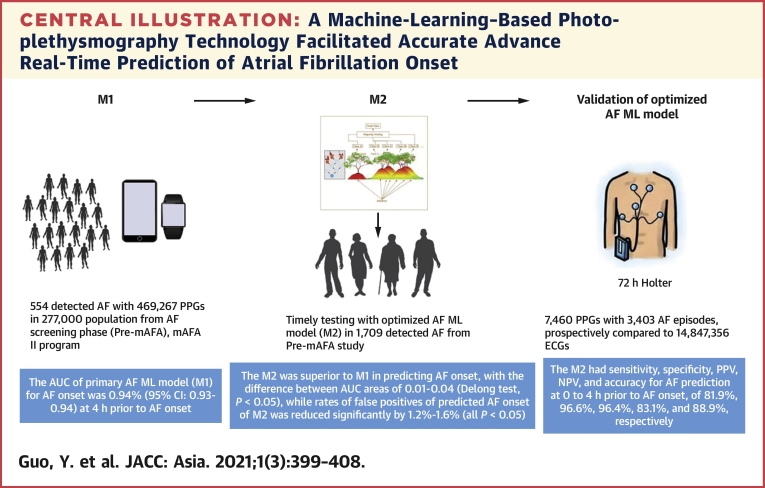
A Machine-Learning–Based Photoplethysmography Technology Facilitated Accurate Advance Real-Time Prediction of Atrial Fibrillation Onset AF = atrial fibrillation; AUC = area under the receiver operating characteristic curve; ECG = electrocardiography; mAFA, mobile Health Technology for Improved Screening, Patient Involvement And Optimizing Integrated Care in Atrial Fibrillation; M1 = primary AF ML model; M2 = optimized AF ML model; ML = machine learning; NPV = negative predictive value; PPG = photoplethysmography; PPV = positive predictive value.

## Funding Support and Author Disclosures

This research project was funded by the National Natural Science Foundation of China (8147413) and partly supported by the National Institute for Health Research Global Health Research Group on Atrial Fibrillation Management at the University of Birmingham, United Kingdom. Dr Lip is a consultant for Bayer/Janssen, BMS/Pfizer, Medtronic, Boehringer Ingelheim, Novartis, Verseon, and Daiichi-Sankyo; and is a speaker for Bayer, BMS/Pfizer, Medtronic, Boehringer Ingelheim, and Daiichi-Sankyo; no fees were directly received personally. All other authors have reported that they have no relationships relevant to the contents of this paper to disclose.
