# Supplementary material for: Photoplethysmography-Based Machine Learning Approaches for Atrial Fibrillation Prediction: A Report From the Huawei Heart Study
Source: JACC Asia. 2021 Dec 21;1(3):399–408. doi: 10.1016/j.jacasi.2021.09.004 (PMC9627828; doi:10.1016/j.jacasi.2021.09.004)
Supplement: Supplemental Tables 1–5 and Supplemental Figures 1–6 [file mmc1.docx]

**Supplemental Table 1 False positives of the primary AF ML model and the optimized AF ML model**

| Cutoff point | Test 1 | | | | | | Test 2 | | | | | | | *P1* | *P2* |
| --- | --- | --- | --- | --- | --- | --- | --- | --- | --- | --- | --- | --- | --- | --- | --- |
|  | M1 | | | M2 | | | M1 | | | | M2 | | |  |  |
|  | Sample size, n | False positives, n | False-positive rate,  %, 95% CI | Sample size, n | False positives, n | False-positive rate,  %, 95% CI | | Sample size, n | False positives, n | False-positive rate,  %, 95% CI | Sample size, n | False positives, n | False-positive rate,  %, 95% CI |  |  |
| 0.1 | 119999 | 764 | 0.64  (0.59–0.68) | 119999 | 480 | 0.40  (0.37–0.44) | | 19999 | 567 | 0.47  (0.44–0.51) | 119999 | 360 | 0.30  (0.27–0.33) | <0.001 | <0.001 |
| 0.2 | 119999 | 1392 | 1.16  (1.10–1.22) | 119999 | 814 | 0.68  (0.63–0.73) | | 119999 | 1314 | 1.09  (1.04–1.16) | 119999 | 702 | 0.59  (0.54–0.63) | <0.001 | <0.001 |
| 0.3 | 119999 | 2051 | 1.71  (1.64–1.78) | 119999 | 1234 | 1.03  (0.97–1.09) | | 119999 | 2146 | 1.79  (1.71–1.86) | 119999 | 1140 | 0.95  (0.90–1.01) | <0.001 | <0.001 |
| 0.4 | 119999 | 2810 | 2.34  (2.26–2.49) | 119999 | 1731 | 1.44  (1.38–1.51) | | 119999 | 3075 | 2.56  (2.47–2.65) | 119999 | 1734 | 1.44  (1.38–1.51) | <0.001 | <0.001 |
| 0.5 | 119999 | 3813 | 3.18  (3.08–3.28) | 119999 | 2408 | 2.00  (1.93–2.09) | | 119999 | 4305 | 3.59  (3.48–3.69) | 119999 | 2446 | 2.04  (1.96–2.12) | <0.001 | <0.001 |
| 0.6 | 119999 | 5167 | 4.31  (4.19–4.42) | 119999 | 3373 | 2.81  (2.72–2.91) | | 119999 | 5981 | 4.98  (4.86–5.11) | 119999 | 3545 | 2.95  (2.86–3.05) | <0.001 | <0.001 |
| 0.7 | 119999 | 7194 | 5.99  (5.86–6.13) | 119999 | 4806 | 4.00  (3.90–4.12) | | 119999 | 8580 | 7.15  (7.01–7.30) | 119999 | 5230 | 4.36  (4.24–4.48) | <0.001 | <0.001 |
| 0.8 | 119999 | 10708 | 8.92  (8.76–9.09) | 119999 | 7621 | 6.36  (6.21–6.49) | | 119999 | 12954 | 10.79  (10.62–10.9) | 119999 | 8333 | 6.94  (6.80–7.09) | <0.001 | <0.001 |

*P1: The primary AF ML model (M1) compared with the optimized AF ML model (M2) with Test 1; P2: The primary AF ML model (M1) compared with the optimized AF ML model (M2) with Test 2. Test 1: Testing with 30,640 PPG signals for AF and 89,359 PPG signals for non-AF of 138 detected AF by PPG algorithm, further confirmed by doctors; Test 2: testing with 30,640 PPG signals for AF and 89,359 PPG signals for non-AF from a total 554 detected AF.

**Supplemental Table 2 Baseline characteristics of the validated cohort compared with 72-h Holter ECG**

|  | Subjects (n=50) |
| --- | --- |
| Female sex, n (%) | 20 (40.0%) |
| Age, mean (SD) | 66.9 (12.0) |
| CHA_2_DS_2_-VASc, mean (SD) | 1.69 (1.34) |
| HAS-BLED, mean (SD) | 0.86 (0.73) |
| Palpitation, n (%) | 7 (14.0%) |
| CAD, n (%) | 13 (26.0%) |
| Hypertension, n (%) | 12 (24.3%) |
| PVD, n (%) | 7 (14.0%) |
| Diabetes, n (%) | 6 (12.0%) |
| OSAS, n (%) | 5 (10.0%) |
| Heart failure, n (%) | 3 (6.0%) |
| Prior ischemic stroke, n (%) | 3 (6.0%) |

*Reported as N (%) unless specified otherwise. SD: standard deviation. CAD: coronary artery disease. PVD: peripheral vascular disease. OSAS: obstructive sleep apnea syndrome.

**Supplemental Table 3 ‘Real’ events of false positives of AF prediction 0 to 4 h prior to AF onset by the optimized AF ML model**

| Cutoff point | Fast/slow heart beat | Atrial bigeminy and/or triad | Ventricle bigeminy and/or triad | Atrial flutter | Ventricle premature | Atrial premature beats | Others | The number of false predicted AF, counts | The false-positive rate of predicting AF, % (95%CI) | The rate of atrial bigeminy, triad, and atrial flutter among total false-predicted events, % (95% CI) |
| --- | --- | --- | --- | --- | --- | --- | --- | --- | --- | --- |
| 0.1 | 7 | 14 | 3 | 110 | 1 | 0 | 29 | 164 | 3.00  (2.58–3.48) | 0.76  (0.68–0.81) |
| 0.2 | 7 | 24 | 3 | 143 | 1 | 0 | 30 | 208 | 3.80  (3.33–4.34) | 0.80  (0.74–0.85) |
| 0.3 | 9 | 31 | 3 | 152 | 1 | 0 | 30 | 226 | 4.13  (3.64–4.69) | 0.81  (0.75–0.85) |
| 0.4 | 10 | 64 | 3 | 158 | 1 | 0 | 31 | 267 | 4.88  (4.34–5.49) | 0.83  (0.78–0.87) |
| 0.5 | 11 | 99 | 0 | 160 | 4 | 0 | 31 | 305 | 5.58  (5.00–6.22) | 0.85  (0.80–0.88) |
| 0.6 | 16 | 159 | 3 | 164 | 1 | 0 | 31 | 375 | 6.86  (6.22–7.56) | 0.86  (0.82–0.89) |
| 0.7 | 18 | 255 | 3 | 164 | 1 | 1 | 31 | 473 | 8.65  (7.93–9.42) | 0.89  (0.86–0.91) |
| 0.8 | 20 | 433 | 3 | 166 | 1 | 1 | 31 | 655 | 11.98  (11.14–12.86) | 0.92  (0.89–0.93) |
| 0.9 | 39 | 947 | 3 | 167 | 1 | 1 | 46 | 1204 | 22.01  (20.94–23.13) | 0.93  (0.91–0.94) |

*There were no recorded episodes of atrial tachycardia or ventricular tachycardia.

**Supplemental Table 4 The accuracy of the optimized AF ML model for AF onset using 3,403 AF episodes by 72-h Holter ECG**

|  | Sensitivity | Specificity | PPV | NPV | Accuracy |
| --- | --- | --- | --- | --- | --- |
| 1 h | 0.81 | 0.94 | 0.94 | 0.81 | 0.87 |
| 2 h | 0.78 | 0.95 | 0.95 | 0.77 | 0.86 |
| 3 h | 0.75 | 0.95 | 0.96 | 0.73 | 0.84 |
| 4 h | 0.73 | 0.96 | 0.96 | 0.70 | 0.82 |

*****PPV: positive predictive value. NPV: negative predictive value.

**Supplemental Table 5 Definitions of features**

| **Features** | **Definition** |
| --- | --- |
| **Heart rate** |  |
| MinHR | Minimum value of RR |
| MeanHR | Mean RR |
| MedianHR | Median RR |
| SkRR | $sk\_RR=E\left[ {(\frac{X-mean}{SDNN})}^{3} \right]$ |
| **Heart rate variability** |  |
| RR | R-R intervals: the time between two successive heart beats |
| NN | N-N interval: the time interval between R peaks, distinguishing from “normal” R-R intervals. |
| CVRR | $CVrr=\frac{1}{mean}\sqrt{\frac{\sum_{i=1}^{n} {(x_{i}-mean)}^{2}}{n-1}}$ |
| SDNN | $SDNN=\sqrt{\frac{\sum_{i=1}^{n} {(x_{i}-mean)}^{2}}{n-1}}$ |
| SDR | $SDratio= \frac{\sqrt{0.5*{RMSSD}^{2}}}{\sqrt{2*{SDNN}^{2}-0.5*{RMSSD}^{2}}}$  $RMSSD=\sqrt{\frac{\sum_{i=1}^{n-1} {(x_{i+1}-x_{i})}^{2}}{n-1}}$  $ShE=\sum_{i=1}^{n} 2x_{i}^{2}{log}_{2}x_{i}$ |
| pNN50 | Number of RRI> 50 ms/HR |
| **User-defined features** |  |
| AFprob | The probability of AF occurrence with the available real-time PPG signals by the ML model |
| **Mathematical features** |  |
| Sample entropy | $\boldsymbol{Samp}\mathbf{En}\left( \mathbf{m,r,L} \right)\mathbf{=-ln}\frac{\boldsymbol{w}^{\boldsymbol{m+1}}\left( \boldsymbol{r} \right)}{\boldsymbol{w}^{\boldsymbol{m}}\left( \boldsymbol{r} \right)}$  **where**  **(1)**  $\boldsymbol{w}^{\boldsymbol{m}}\left( \boldsymbol{r} \right)\mathbf{=}\frac{\boldsymbol{1}}{\boldsymbol{L-m}}\sum_{\boldsymbol{i}\boldsymbol{=}\boldsymbol{1}}^{\boldsymbol{L-m}} \frac{\boldsymbol{Bi}}{\boldsymbol{L-m-1}}$  **(2)**  **X(i)** is a time series **X(i)=x(1), x(2),…, x(L)** with **L** data points  **(3)**  **Xm(i) = {x(i), x(i + 1), …, x(i + m − 1)}**, i = 1, 2, …, L − m + 1  **(4)**  The distance **d** between the vectors Xm(i) and Xm(j) is:  **d[Xm(i), Xm(j)] = max (\|x(i + l) − x(j + l)\|), l = 0, 1, …, m**  **(5)**  **r = kσ** where σ is the standard deviation of L data points, and k is a constant.  **(6)**  **Bi** is defined as the number of Xm(j) satisfying d[Xm(i), Xm(j)]≤**r**, i≠j. The number of reconstituted vectors is **L-m** |
| Pointcare stepping | $\boldsymbol{PoincareStp}\mathbf{=}\frac{\sum_{\boldsymbol{i=1}}^{\boldsymbol{L}} \boldsymbol{x}_{\boldsymbol{i}}^{\frac{\boldsymbol{1}}{\boldsymbol{2}}}}{\boldsymbol{(L-2)*mean}}$  **where**  $\boldsymbol{mean=}\frac{\sum_{\boldsymbol{i=1}}^{\boldsymbol{L}} \boldsymbol{x}_{\boldsymbol{i}}}{\boldsymbol{L}}$  **X(i)** is a time series **X(i)=x(1), x(2),…, x(L)** with **L** data points |
| Frequency domain | **FreqDom = 𝐿𝐹/𝐻𝐹**  **where**  **(1)**  Low frequency (**LF**) is the ITU designation for radio frequencies in the range of 30–300 kHz. Since its wavelengths range from 10–1 km, it is also known as the kilometer band or kilometer wave.  **(2)**  High frequency (**HF**) is the ITU designation for the range of radio frequency electromagnetic waves (radio waves) between 3 and 30 MHz. It is also known as the decameter band or decameter wave as its wavelengths range from 1 to 10 decameters (10 to 100 meters) |
| Shannon entropy | $\mathbf{ShannonEn=}\boldsymbol{-}\sum_{\boldsymbol{i}\boldsymbol{=}\boldsymbol{1}}^{\boldsymbol{n}} \boldsymbol{p}\left( \boldsymbol{x}_{\boldsymbol{i}} \right)\boldsymbol{log}_{\boldsymbol{2}}\boldsymbol{p(}\boldsymbol{x}_{\boldsymbol{i}}\boldsymbol{)}$  **where** $\boldsymbol{p}$ is probability mass function of random variable $\boldsymbol{x}$, and log to base 2 |
| Approximate entropy | $\boldsymbol{Ap}\mathbf{En}\left( \mathbf{m,r,L} \right)\mathbf{=}\boldsymbol{w}^{\boldsymbol{m}}\left( \boldsymbol{r} \right)\mathbf{-}\boldsymbol{w}^{\boldsymbol{m+1}}\left( \boldsymbol{r} \right)$  **where**  **(1)**  $\boldsymbol{w}^{\boldsymbol{m}}\left( \boldsymbol{r} \right)\mathbf{=}\frac{\boldsymbol{1}}{\boldsymbol{L-m+1}}\sum_{\boldsymbol{i}\boldsymbol{=}\boldsymbol{1}}^{\boldsymbol{L-m+1}} \boldsymbol{ln}\frac{\boldsymbol{Bi}}{\boldsymbol{L-m+1}}$  **(2)**  **X(i)** is a time series **X(i)=x(1), x(2),…, x(L)** with **L** data points  **(3)**  **Xm(i) = {x(i), x(i + 1), …, x(i + m − 1)}**, i = 1, 2, …, L − m + 1  **(4)**  The distance **d** between the vectors Xm(i) and Xm(j) is:  **d[Xm(i), Xm(j)] = max (\|x(i + l) − x(j + l)\|), l = 0, 1, …, m**  **(5)**  **r = kσ** where σ is the standard deviation of L data points, and k is a constant.  **(6)**  **Bi** is defined as the number of Xm(j) satisfying d[Xm(i), Xm(j)]≤**r**, i≠j. The number of reconstituted vectors is **L-m** |
| Difference | **Diff** $\boldsymbol{=}\frac{\sum_{\boldsymbol{i}\boldsymbol{=0}}^{\boldsymbol{n}} \boldsymbol{\Delta}\mathbf{y(}\boldsymbol{x}_{\boldsymbol{i}}\mathbf{)}}{\sum_{\boldsymbol{i}\boldsymbol{=0}}^{\boldsymbol{n}} \boldsymbol{\Delta}\mathbf{(}\boldsymbol{\Delta}\mathbf{y(}\boldsymbol{x}_{\boldsymbol{i}}\boldsymbol{)}\mathbf{)}}$  **where**  **Δy(x)** = **y(x+1) - y(x)**  **Δ(Δy(x))=Δ(y(x+1) - y(x))=Δy(x+1) - Δy(x)**  **=(y(x+2) - y(x+1)) - (y(x+1) - y(x))**  **=y(x+2) - 2y(x+1) + y(x)** |
| PPG monitoring time | **MoniTime = t**  **where**  t the monitoring time， 0–24 hours |


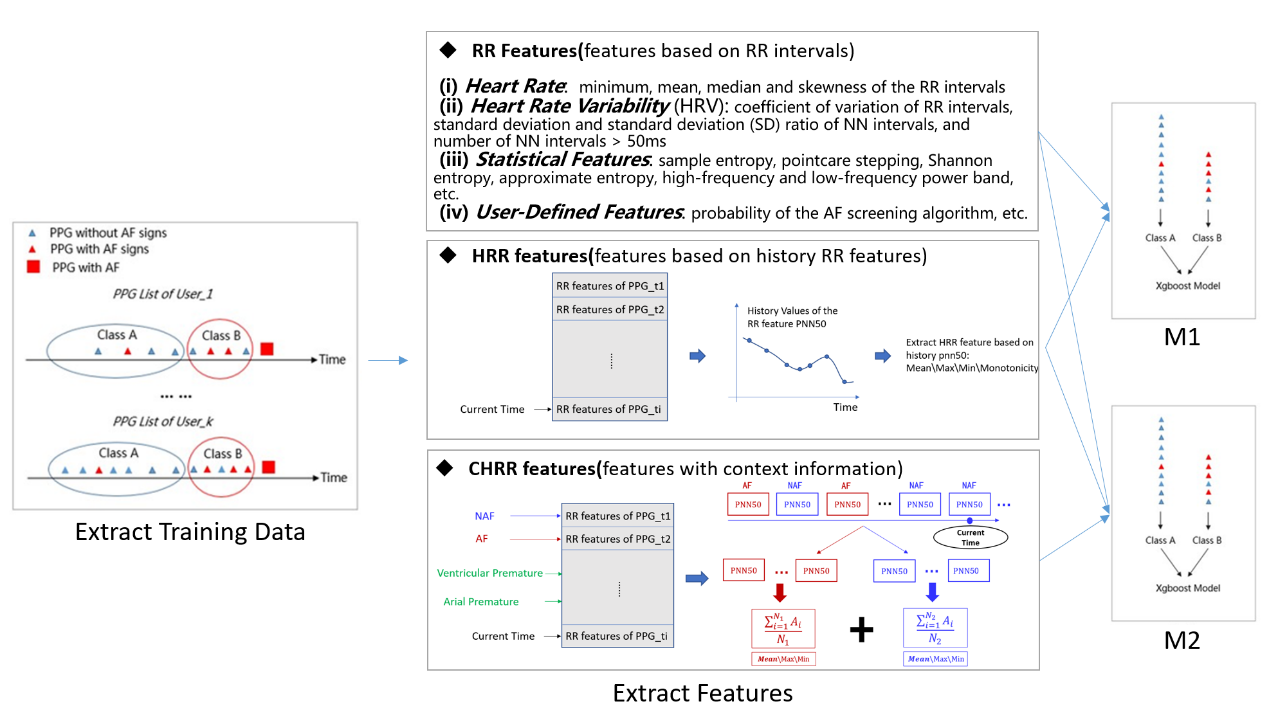


**Supplemental Figure 1 AF ML model architecture.**

**
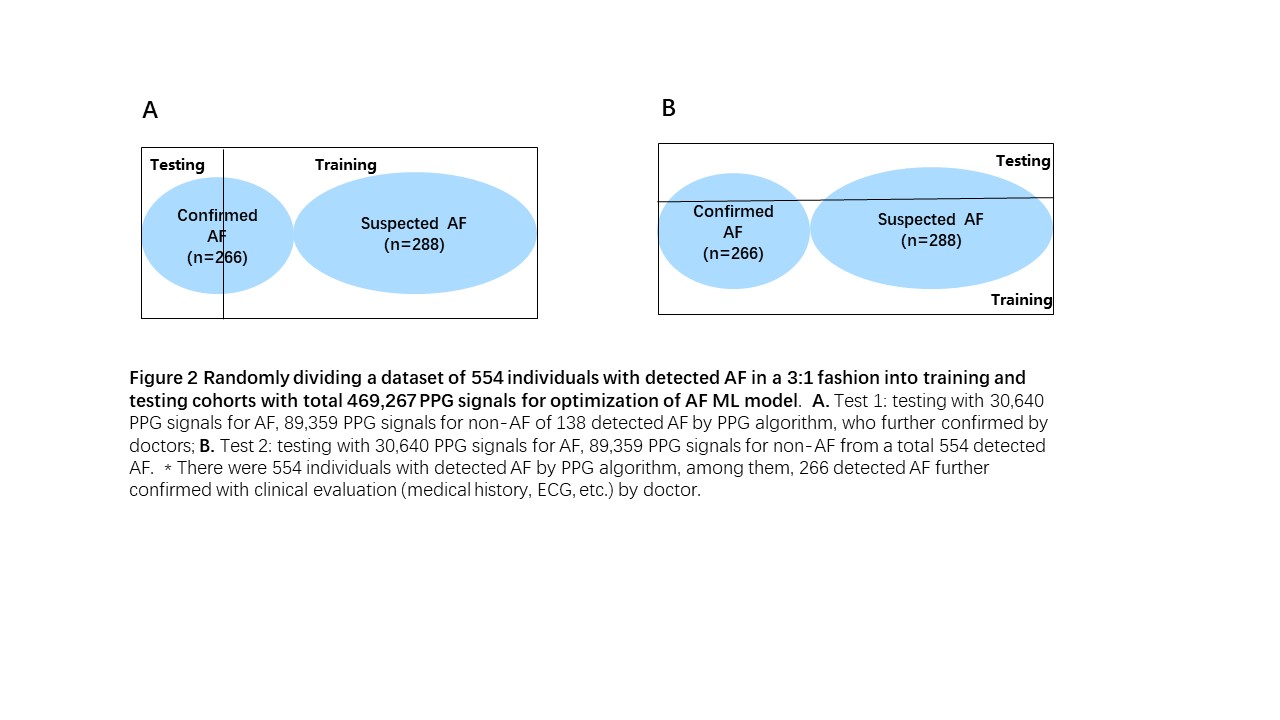
 Supplemental Figure 2 Randomly dividing a dataset of 554 individuals with detected AF in a 3:1 fashion into the training cohort and the testing cohort with total of 469,267 PPG signals for optimization of the AF ML model. A.** Test 1: testing with 30,640 PPG signals for AF and 89,359 PPG signals for non-AF of 138 detected AF by PPG algorithm, further confirmed by doctors. **B.** Test 2: testing with 30,640 PPG signals for AF and 89,359 PPG signals for non-AF from a total of 554 detected AF. *There were 554 individuals with detected AF by PPG algorithm; among them, 266 detected AFs were further confirmed by clinical evaluation (medical history, ECG, etc.) by doctor.


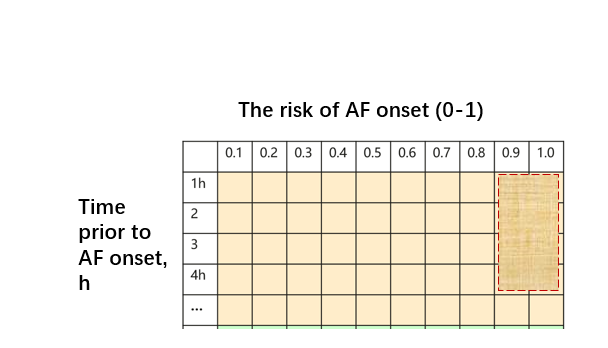


**Supplemental Figure 3 The model of sample size calculation of prospective high-risk validation cohort.**


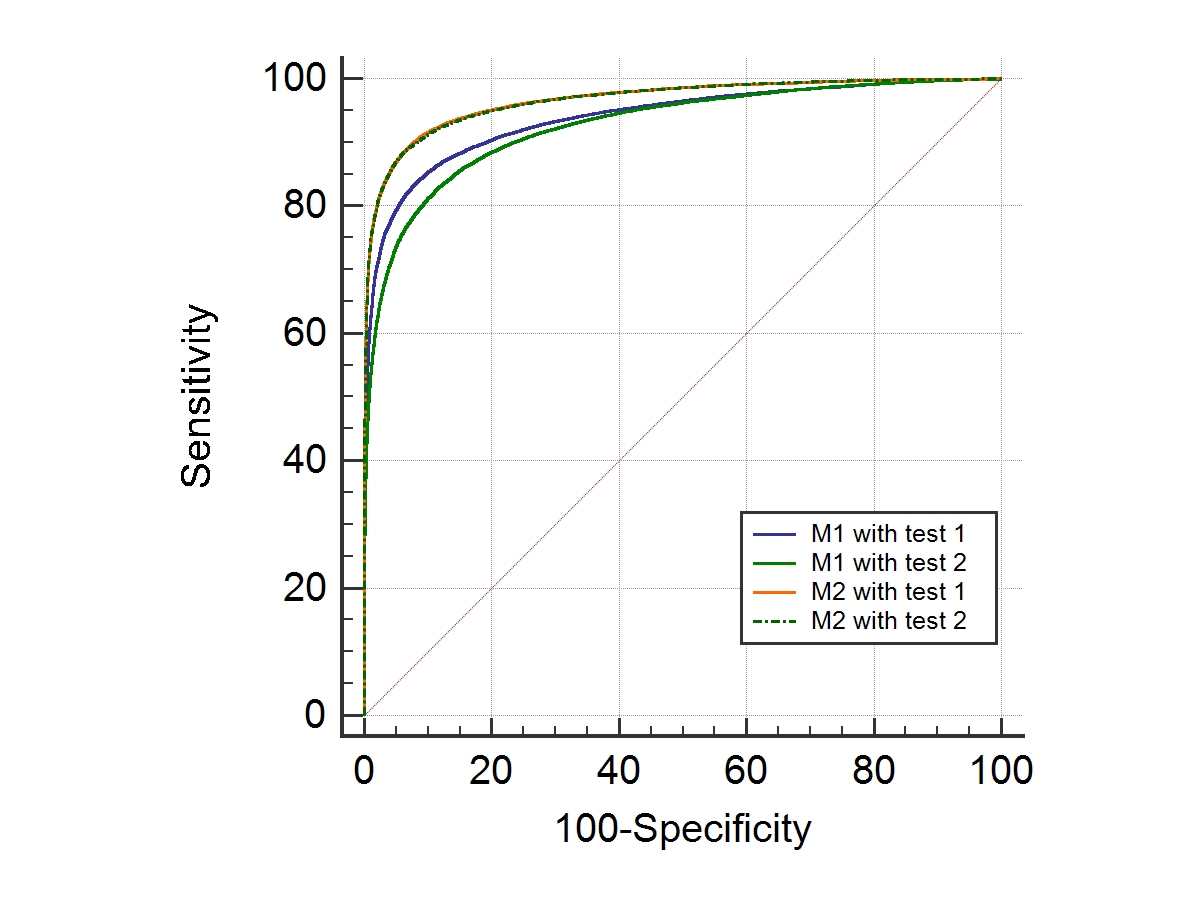


**Supplemental Figure 4 Comparison of ROC curves of the primary AF ML model (M1) and the optimized AF ML model (M2).** The M2 was superior to M1 in predicting AF onset, with the difference between AUC areas of 0.01–0.04, using two randomly split datasets (Delong test, all P <0.05). ROC: receiver operating characteristic curve. Test 1: testing with 30,640 PPG signals for AF and 89,359 PPG signals for non-AF of 138 detected AF by PPG algorithm, further confirmed by doctors. Test 2: testing with 30,640 PPG signals for AF and 89,359 PPG signals for non-AF from a total of 554 detected AF.


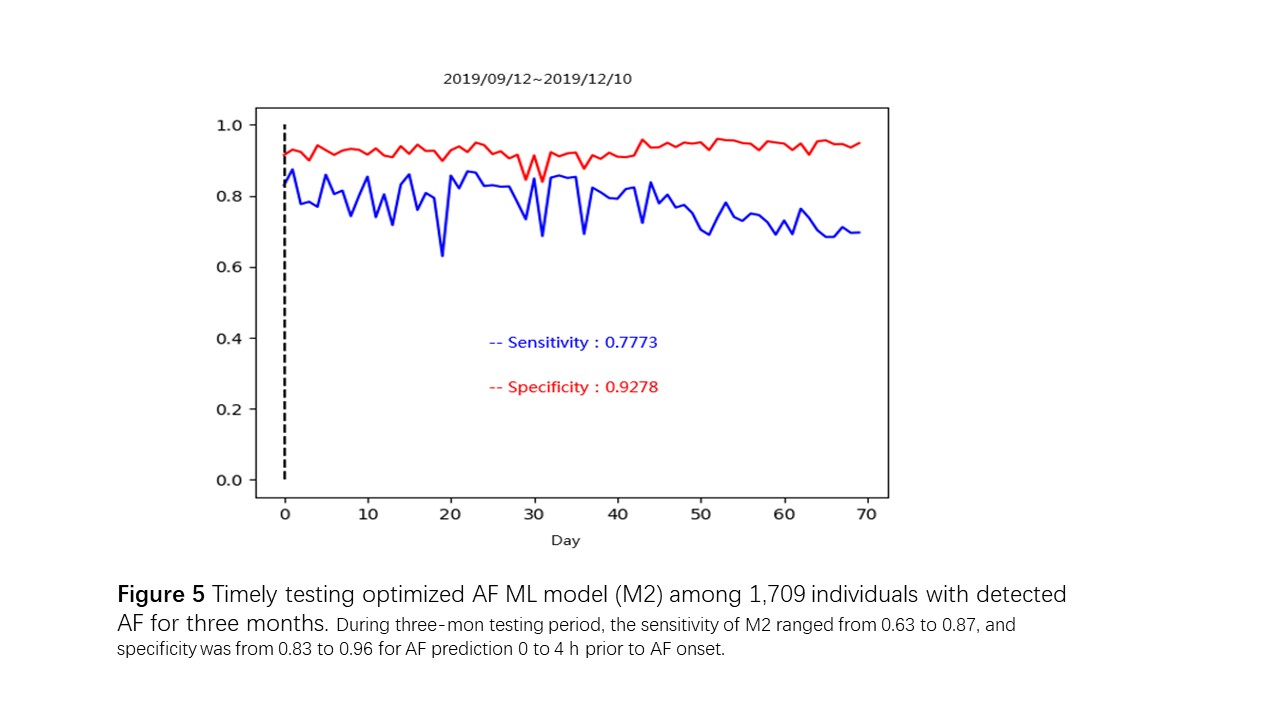


**Supplemental Figure 5 Testing of the optimized AF ML model (M2) among 1,709 individuals with detected AF during 3 months.** During the 3-month testing period, the sensitivity of M2 ranged from 0.63 to 0.87 (average 0.78), and specificity ranged from 0.83 to 0.96 (average 0.93) for AF prediction 0 to 4 h prior to AF onset


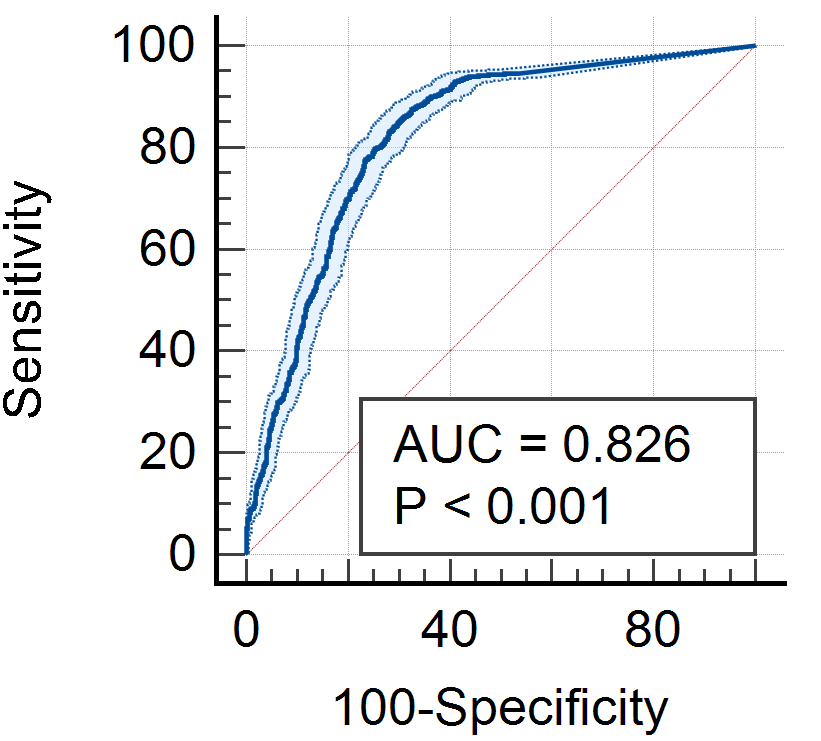


**Supplemental Figure 6 ROC curve of the M2 to predict AF episodes in ECG, Test 4.**
